# Effects of serial and acute enteric-coated sodium bicarbonate supplementation on anaerobic performance, physiological profile, and metabolomics in healthy young men

**DOI:** 10.3389/fnut.2022.931671

**Published:** 2022-08-16

**Authors:** Nihong Zhou, Yongzhao Fan, Xiaoyang Kong, Xiangyu Wang, Junde Wang, Hao Wu

**Affiliations:** ^1^Graduate School, Capital University of Physical Education and Sports, Beijing, China; ^2^Qingdao Shengbang Health Food Co., Qingdao, China; ^3^School of Kinesiology and Health, Comprehensive Key Laboratory of Sports Ability Evaluation and Research of the General Administration of Sport of China, Beijing Key Laboratory of Sports Function Assessment and Technical Analysis, Capital University of Physical Education and Sports, Beijing, China

**Keywords:** sodium bicarbonate supplementation, anaerobic performance, physiological profile, gastrointestinal reactions, metabolomics

## Abstract

**Background:**

Previous studies have reported that sodium bicarbonate ingestion may enhance high-intensity exercise performance and cause severe gastrointestinal distress. However, enteric-coated sodium bicarbonate may reduce gastrointestinal symptoms of sodium bicarbonate after oral administration. This remains to be confirmed. This study aimed to verify the effects of serial and acute enteric-coated sodium bicarbonate supplementation on anaerobic performance, physiological profile, and metabolomics in healthy young men.

**Methods:**

Healthy young males (*n* = 12) ingested 0.2 g/kg body mass of enteric-coated sodium bicarbonate (ES) in serial enteric-coated sodium bicarbonate (SES, continuous ES supplementation for 5 days) and acute enteric-coated sodium bicarbonate (AES, acute ES supplementation before exercise) or a placebo (PL) in a randomized crossover design. After each supplement protocol, the participants completed four Wingate anaerobic tests (WAT). The first three Wingate tests (testing anaerobic capacity) were performed with a 5-min passive recovery between each. After the third Wingate test, participants were required to complete a 50-min recovery followed by a fourth WAT test (testing the recovery of anaerobic capacity after 50-min intervals). Blood lactate (BLA), heart rate (HR), and ratings of perceived exertion (RPE) were measured in all conditions during the test, as was the subjective gastrointestinal–symptoms assessment questionnaire (GSAQ). Mean power (MP) and peak power (PP) were recorded after four WATs. Urine samples were collected before the test and 50 min after the 3rd WAT.

**Results:**

Serial enteric-coated sodium bicarbonate supplementation improved anaerobic capacity in the third bout of WATs, as observed based on an increase in mean power (SES vs. PL (613 ± 57 vs. 542 ± 64 W), *P* = 0.024) and peak power (SES vs. PL (1,071 ± 149 vs. 905 ± 150 W), *P* = 0.016). Acute ES supplementation did not affect anaerobic capacity. The occurrence of gastrointestinal symptoms after enteric-coated sodium bicarbonate supplementation was minimal and no difference compared to placebo in the current study. In particular, serial enteric-coated sodium bicarbonate supplementation had no gastrointestinal side effects before the test. The AES and SES groups had a trivial effect on blood lactate compared to the PLA group. There was no significant difference in HR and RPE among the three groups. Based on targeted metabolomics analysis, the 50 min after the third WAT, the levels of lactate (*P* < 0.001), L-Malic acid (*P* < 0.05), and oxaloacetate (*P* < 0.05) were significantly higher in the SES group than in the PL group. Compared with the AES group, the levels of lactate and fumarate in the SES group were significantly increased (*P* < 0.05).

**Conclusions:**

Our study indicates that serial enteric-coated sodium bicarbonate supplementation positively improves anaerobic performance among healthy young men. However, acute ingestion of enteric-coated sodium bicarbonate did not improve anaerobic exercise performance. Either with serial or acute supplementation doses, enteric-coated sodium bicarbonate produced fewer gastrointestinal symptoms and no difference compared to placebo, especially with no gastrointestinal side effects after serial supplementation. Serial and acute supplementation of enteric-coated sodium bicarbonate might tend to promote lactate clearance. Furthermore, serial enteric-coated sodium bicarbonate ingestion may cause changes in the metabolism of lactate, L-Malic acid, oxaloacetate, and fumarate 50 min after exercise, which presumably may promote the tricarboxylic acid cycle and lactate clearance.

## Introduction

Sodium bicarbonate (NaHCO_3_) is an ergogenic aid and has been well-documented. When working muscle cells have a limited supply of oxygen during high-intensity exercise, lactate and hydrogen ions (H^+^) accumulate as a result of increased glycolysis (1). The metabolic acidosis caused by the increase in H^+^ concentration and the concomitant reduction in sarcoplasmic pH is considered to be the main causes of muscular fatigue during high-intensity exercise (2, 3). The buffering systems' ability to remove H^+^ from muscle cells is critical for maintaining muscular contractility (4). As a result, strategies to buffer the exercise-induced intracellular and extracellular acidosis are required. As an extracellular buffering agent, NaHCO_3_ improves endogenous bicarbonate buffering capacity by inducing significant, albeit transient, increases in extracellular bicarbonate (5). The increased H^+^ gradient on monocarboxylate transporters (MCTs) that carry lactate across muscle cell membranes, induced by sodium bicarbonate ingestion, augments the efflux of lactate and H^+^ from muscle cells into circulation (6), which is essential for regulating pH concentrations and supporting metabolic functions.

Studies have investigated the ergogenic effects of sodium bicarbonate loading in a range of activities, including cycling (7, 8), running (9), swimming (10), and Wrestling (11), with differing results. Most empirical studies support sodium bicarbonate as beneficial for different types, durations, and intensities of exercise performance (12, 13). However, there are claims that sodium bicarbonate ingestion has no beneficial effects on performance (14, 15). Furthermore, ingesting sodium bicarbonate can cause gastrointestinal (GI) side effects, and studies have shown that some people experience severe symptoms during the pre-exercise (16). Although the influence of GI discomfort on performance is ambiguous, symptoms like vomiting and diarrhea may be a considerable problem for athletes and coaches. To relieve GI discomfort, researchers are testing new intake tactics. Applying an enteric coating (Polymeric-coated compounds) can resist gastric degradation and reduce GI symptoms provoked by acid-sensitive compounds, such as NaHCO_3_ (17). However, whether taking enteric-coated sodium bicarbonate affects the overall performance of the anaerobic exercise is still unclear.

Although some studies have shown that serial loading can improve athletic performance in athletes (18–21), we only know that a few studies have compared serial loading with the more commonly used acute loading protocols in athletes (7). A recent study indicates that chronic and acute sodium bicarbonate supplementation positively supports discipline-specific performance among field hockey athletes (22). In contrast, another study indicates that acute and chronic loading of NaHCO_3_ does not improve 200-m swim performance in highly trained male swimmers (23). Given the conflicting results of previous research and the lack of more data to support enteric-coated sodium bicarbonate (ES), the effectiveness of serial and acute enteric-coated sodium bicarbonate loading requires further investigation.

Metabolomics is a rapidly evolving field of life science that uses advanced analytical chemistry techniques and sophisticated statistical methods to characterize the metabolome comprehensively (24). Metabolomics is the profiling of metabolites in biofluids, cells, and tissues. The rapid growth in metabolomics leads to a renewed interest in metabolism and the role that small molecule metabolites play in many biological processes. Targeted metabolomics identifies and quantifies a small subset (50–500) of compounds within the metabolome (25), which is ideal for hypothesis testing and biomarker detection. It focuses on analyzing specific metabolic pathways and specific effects of certain nutrients. However, metabolomics has not been applied to the study of the effects of enteric-coated sodium bicarbonate supplementation on metabolic mechanisms in humans.

Therefore, the primary aim of this study was to investigate the effects of both serial and acute enteric-coated sodium bicarbonate supplementation on anaerobic performance and the physiological profile of high-intensity exercise. And the target metabolome based on the MRM method was used to analyze the specific metabolic effects. We hypothesized that serial and acute enteric-coated sodium bicarbonate supplementation would not cause gastrointestinal discomfort. Serial enteric-coated sodium bicarbonate supplementation would improve anaerobic power indices and enhance performance to a greater degree than acute enteric-coated sodium bicarbonate supplementation.

## Methods

### Participants

The sample size for this study was determined with the G^*^power 3.1.9.7 software. A power analysis revealed that a minimum of 8 participants in each group was required to achieve a statistical power of 0.85, with effect sizes of f (V) = 0.25 and α = 0.05. Twelve healthy young male participants were recruited to participate in this study (Table 1). All participants volunteered to participate in the study after being informed verbally and in writing of the nature and risks associated with the study. The participants were free from chronic and other diagnosed diseases. They did not experience discomfort or injury, did not take any nutritional supplements or medications, and did not drink beverages other than water. Ethical approval was obtained by the ethics committee of Capital University of Physical Education and Sports (Review approval number: 2021A42; Beijing, China), and all participants gave written informed consent to participate in the study.

**Table 1:** Baseline data of participants (mean ± SD).

**Sex**	**Number**	**Age (Years)**	**Height (cm)**	**Body mass (kg)**	**BMI**	**Fat-free mass (kg)**	**Body fat (%)**
Male	12	24.00 ± 1.54	178.42 ± 4.14	73.98 ± 5.51	23.25 ± 1.63	62.56 ± 4.74	15.30 ± 5.12

### Study design

This study was performed in a randomized crossover design. Each participant completed three trials at maximum speed under three experimental conditions: serial enteric-coated sodium bicarbonate, acute enteric-coated sodium bicarbonate, and placebo (containing cornstarch). The supplementation dose for this study was 0.2 g/kg BM. For single-dose supplementation protocols, 0.2 g/kg BM of sodium bicarbonate seems to be the minimum dose required to experience improvements in exercise performance (26). Each experimental condition was separated by at least 7 days after completion to allow residual enteric-coated NaHCO_3_ to be washed off (7, 21, 27). And all tests were performed at the same time of day to minimize circadian variation. Wingate anaerobic test (WAT) was adopted in this trial. The Wingate anaerobic test is commonly used to evaluate high-intensity exercise performance, including “all-out” cycling for 30 s on a cycling ergometer (28). Then the participants performed a standardized warm-up at 60 W for 5 min. And the participants performed 4 × WATs as an indicator of instantaneous power production.

**Figure 1:**
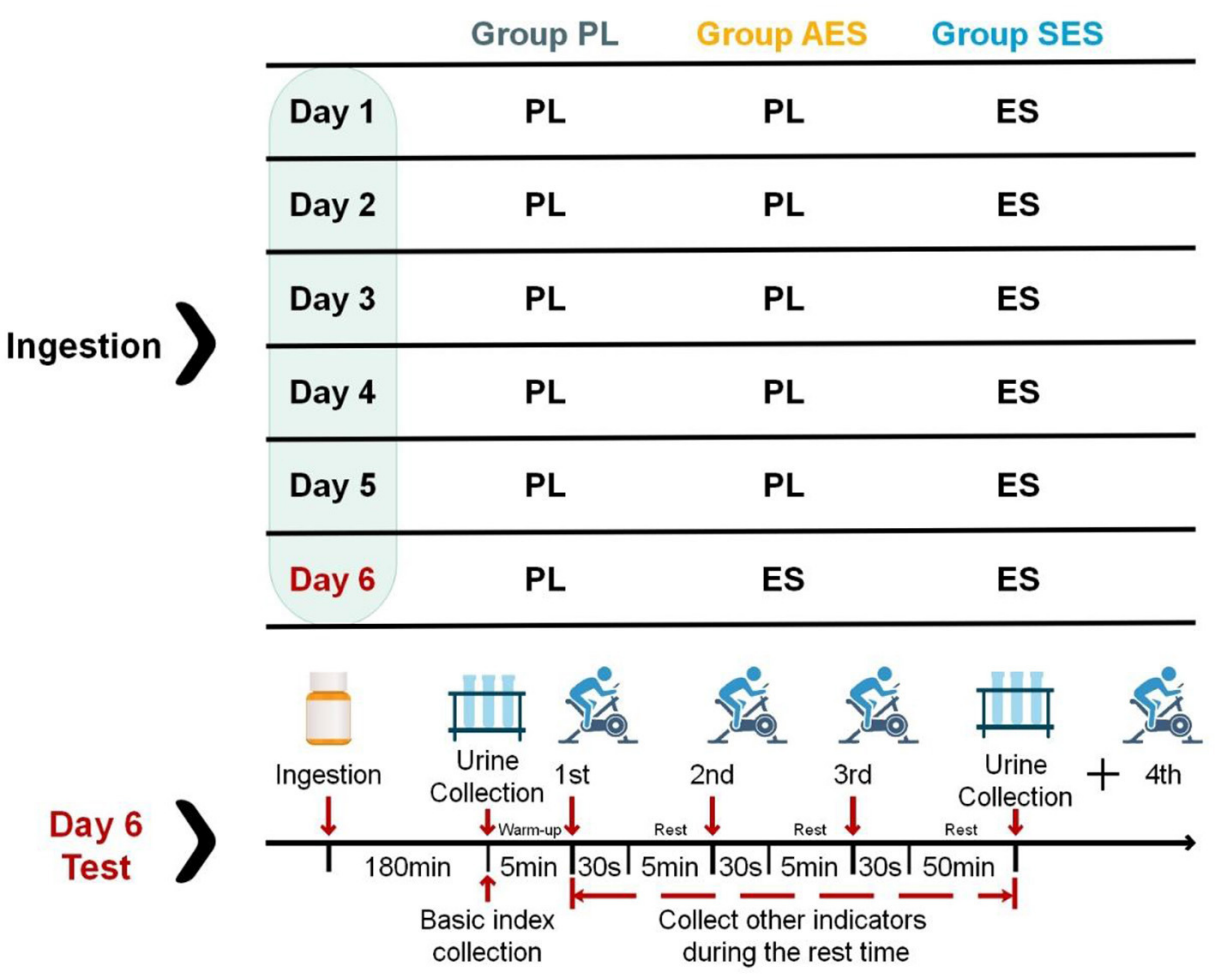
Experimental design for the three trials: placebo group (PL), acute enteric-coated sodium bicarbonate group (AES), and serial enteric-coated sodium bicarbonate group (SES). The ES doses on days 1–5 were taken in equal amounts at breakfast, lunch, and dinner. All the doses were taken on day 6 (pre-test) commenced 180 min before test time.

In addition, the first three WATs were undertaken, with 5 min of passive recovery between each one. After the third WAT, the participants were asked to stop for 50 min to recover and then sprint for the fourth WAT. This experimental exercise protocol was to test the effects of SES or AES intake on anaerobic exercise capacity through the first three WATs. The effect of SES or AES intake on recovery of anaerobic exercise capacity was tested by performing the fourth WAT after a third 50-min interval. During the test, participants were only allowed to drink 200 ml of water and were not allowed to eat any other food before the third WAT. After the third WAT (i.e., within 50 min of the third interval), no water or food was allowed to be consumed as this may affect the urine sample collection. Participants were asked to sit and remain as still as possible during the interval, to avoid affecting the index measurements due to excessive movement. All intervals were passive recovery sessions with standardized monitoring by the researchers (wearing a heart rate monitor throughout). Figure 1 shows the flow chart of the experiment.

### Experimental protocol

Participants were informed of the experimental procedure during their first visit to the laboratory and were simulated throughout the experimental steps in order to familiarize them with the procedure. Prior to each trial, participants were given instructions on the procedure. The experimental design for the three trials was: serial enteric-coated sodium bicarbonate (SES), acute enteric-coated sodium bicarbonate (AES), and placebo (PL). The SES trial required the participants to ingest a 0.2 g kg^−1^ body mass dose of enteric-coated sodium bicarbonate, to be taken in three equal amounts throughout the day, for 5 days (completing their last dose at 7 p.m. the night before testing). On the day of the test for the SES trial, the participants ingested an acute placebo dose, commencing 180 min before the test. In the AES trial, the participants ingested a 0.2 g·kg^−1^ body mass dose of enteric-coated sodium bicarbonate, commencing 180 min before their test time. The AES trial preceded 5 days of placebo supplementation to blind the participants to which condition they were trialing. The PL doses on days 1–5 were taken in equal amounts throughout the day (at breakfast, lunch, and dinner) in the PL trial. All the doses of the placebo taken on day 6 (pre-test) commenced 180 min before test time. Participants were asked to keep their normal schedule on the day before all interventions and to avoid consuming, during the study, carbonated drinks, alcohol, caffeine, or other substances that could affect the Wingate test, blood, and urine test results. Participants were also asked to avoid any strenuous exercise for 48 h before the test.

### Anaerobic exercise tests

Upon arrival at the laboratory on the day of experiments per group, baseline (pre-test) samples were taken 180 min after supplement intake. Participants selected a preferred handlebar and saddle position before the test, then replicated it for all other experimental trials. Following a 5-min warm-up, participants started performing four Wingate anaerobic tests with a Monark 894E (Ergomedic 894E, Sweden). Participants were instructed to complete each test as fast as possible, and the resistance factor was set at 0.075 kg kg^−1^ BW. Participants are asked to sprint as fast as they can while performing the Wingate test. During the 30-s maximum ride, the participants were asked to keep their hips off the seat and were given constant verbal encouragement and time cues to help them perform at their maximum capacity during the test. The peak and mean power outcomes were calculated via Monark Anaerobic Testing software after every test.

### Physiological and perceptual measures

The participants had the index collection to establish basal measurements [blood lactate (BLA), heart rate (HR), ratings of perceived exertion (RPE), and gastrointestinal–symptoms assessment questionnaire (GSAQ)] before the first WAT. BLA concentration was collected from the participants ear lobes pre- and post-test. The blood lactate samples were tested via the h/p/cosmos Sirius^®^ lactate test meter (manufacturer: SensLab GmbH, Germany; accuracy: ±3 %, minimum standard deviation: ±0.2 mmol/L). HR was continuously monitored using a Polar RS800CX telemetry heart rate monitor (manufacturer: Polar, Finland) during the test for analyzing the heart rate. RPE was measured with the Borg 15-point scale during the test, ranging from 6 (very, very light) to 20 (very, very heavy) (29). Before the test, immediately after the third WAT, and 50 min after the third WAT, the participants completed an adapted gastrointestinal–symptoms assessment questionnaire to measure gastrointestinal (GI) side effects (30). The GSAQ was describing possible GI side effects. At the same time, to score the severity of these symptoms, the numeric rating scale (NRS) was utilized (from 1 to 10, with 0 reflecting no GI distress at all and 10 being the most severe GI distress imaginable).

### Detection method of metabolomics

Targeted metabolomics adopts MRM principles. Selective Response/Multi-Reaction Monitoring (SRM/MRM) for the targeted and specific detection and analysis of specific metabolite groups concerning standards and absolute quantification of the target metabolites, with high specificity, sensitivity, and accuracy.

Urine samples were collected before the test and 50 min after the 3rd WAT. The samples were stored in an −80°C refrigerator for analysis. The samples were removed at −80°C and slowly dissolved at 4°C. Then each group of samples was added to 1,000 μl of pre-cooled methanol acetonitrile solution (1:1, v/v), vortexed for the 60 s, left for 1h at −20°C to precipitate the protein, centrifuged at 14,000 rcf for 20 min at 4°C, freeze-dried the supernatant, and stored the samples at −80°C. Analyses were performed using a UHPLC (1,290 Infinity LC, Agilent Technologies) coupled with a QTRAP (AB Sciex 5500).

## Statistical analysis

Statistical analyses were completed using IBM SPSS Statistics version 26.0. The Shapiro–Wilk normality test was applied to assess the normality of distribution. The descriptive data are presented as mean ± SD. For repeated measures, pre-test and post-test data (MP, PP, BLA, HR, RPE) were analyzed using two-factor (condition × time) ANOVA. The metabolomics sample data was extracted using Multiquant software for peak area and retention time. Retention times were corrected using standards of energy metabolites, and metabolite identification was performed. The statistical significance level was set at *P* **<** 0.05.

## Results

### Anaerobic exercise performance

**Figure 2:**
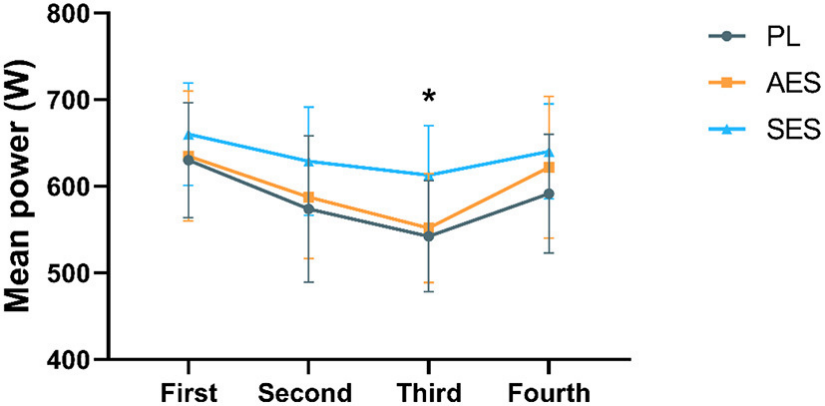
Mean power results of each WAT. * Indicates a significant difference between the serial enteric-coated group and the placebo group (*p* < 0.05).

**Figure 3:**
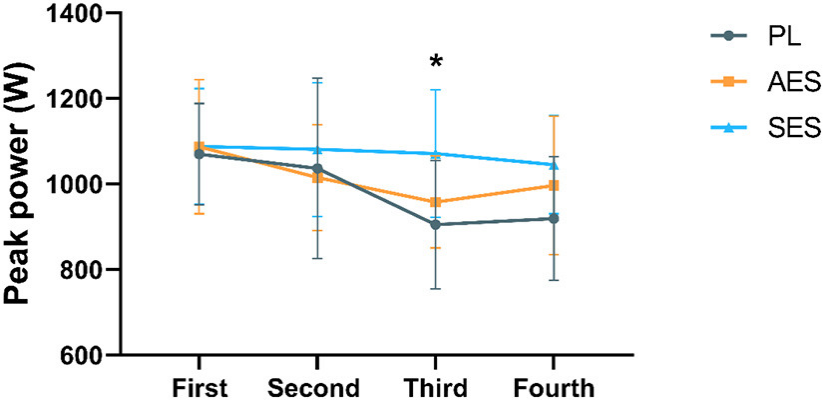
Peak power results of each WAT. * Indicates a significant difference between serial enteric-coated group and placebo group (*p* < 0.05).

Figures 2, 3 show the mean and peak power output and the effect sizes values for all four exercise bouts in three interventions. There was a significant main effect of time on MP (*F*_(2.552, 84.229)_ = 34.550, *P* = 0.000, η^2^ = 0.511) and PP [*F*_(2.852, 94.119)_ = 6.080, *P* = 0.001, η^2^ = 0.156], but no interaction effect between condition and time [MP: *F*_(5.105, 84.229)_ = 1.534, *P* = 0.187, η^2^ = 0.085; PP: *F*_(5.704, 94.119)_ = 1.237, *P* = 0.295, η^2^ = 0.070]. Serial enteric-coated sodium bicarbonate supplementation substantially improved performance in the third WAT but not in the other WATs (Figures 2, 3). MP and PP were significantly higher at SES compared to PL in the third WAT [MP: SES vs. PL (613 ± 57 vs. 542 ± 64 W), *P* = 0.024; PP: SES vs. PL (1,071 ± 149 vs. 905 ± 150 W), *P* = 0.016]. There was no significant effect in MP and PP between the three conditions (MP: *F*_(2, 33)_ = 2.173, *P* = 0.130, η^2^ = 0.116; PP: *F*_(2, 33)_ = 2.245, *P* = 0.122, η^2^ = 0.120).

### Blood lactate responses

**Figure 4:**
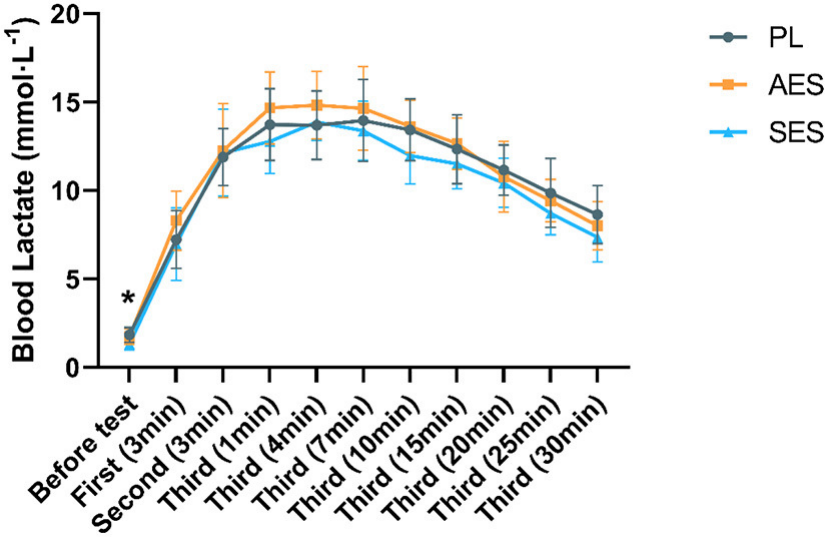
Blood lactate concentration for exercise tests. Values are means ± SD. *Indicates a significant difference between the serial enteric-coated group and the placebo group (*p* < 0.05). First (N min): N minute following the first WAT; Second (N min): N minute following the second WAT; Third (N min): N minute following the third WAT.

BLA had a significant main effect in time [*F*_(5.482, 180.895)_ = 272.148, *P* = 0.000, η^2^ = 0.892], but the main effect of BLA between groups was not significant (*F*_(2, 33)_ = 2.371, *P* = 0.109, η^2^ = 0.126), and there was no condition × time interaction [*F*_(10.963, 180.895)_ = 1.075, *P* = 0.384, η^2^ = 0.061]. BLA was significantly lower in the SES trial compared with the placebo before the test [SES vs. PL, (1.33 ± 0.34 mmol L^−1^ vs. 1.84 ± 0.42 mmol L^−1^), *P* = 0.013]. There was a significant effect of time on the tests. In the three conditions, BLA concentration rose progressively from pre-test to 4 min after the third WAT, after which blood lactate declined during the third WAT interval (Figure 4).

**Figure 5:**
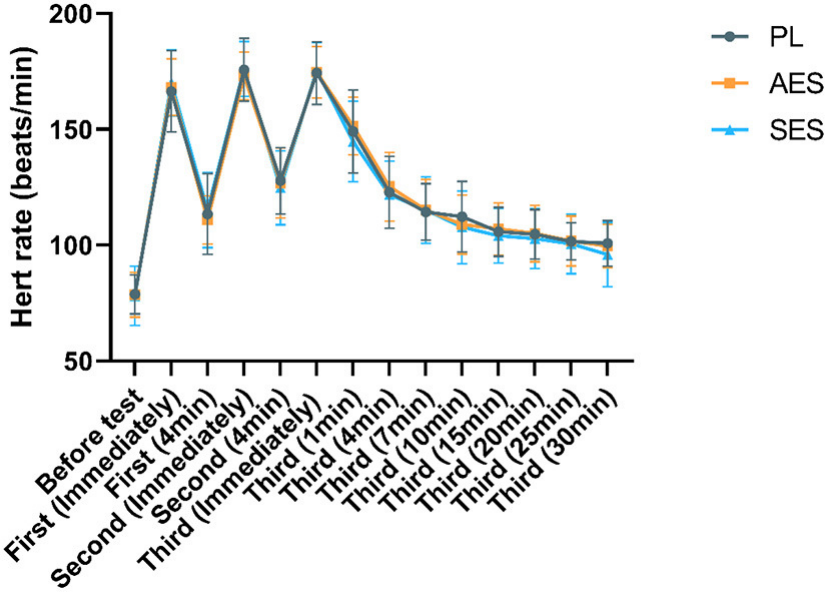
HR responses during three groups of experiments. Values are means ± SD. First (N min): N minute following the first WAT; Second (N min): N minute following the second WAT; Third (N min): N minute following the third WAT.

Heart rate analysis results related to the test are shown in Figure 5. HR gradually increased throughout the experiment [*F*_(5.121, 169.006)_ = 553.417, *P* = 0.000, η^2^ = 0.944]. Although no significant differences were shown between conditions [F _(2, 33)_ = 0.037, *P* = 0.964, η^2^ = 0.002], no significant condition × time interactions were observed [*F*_(10.243, 169.006)_ = 0.600, *P* = 0.816, η^2^ = 0.035]. HR was not significantly different after SES supplementation (Figure 5). Similarly, AES did not affect HR, with no difference observed among the three groups (all *p* > 0.05).

### Perceptual responses

**Figure 6:**
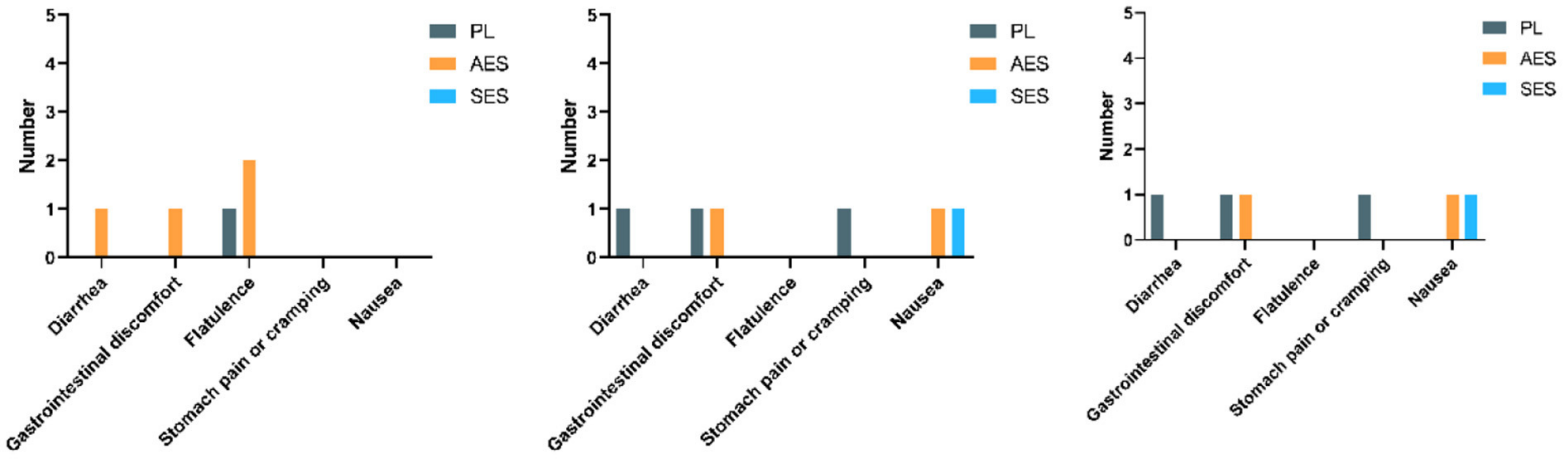
Gastrointestinal–symptoms assessment questionnaire results before exercise, immediately, and 50 min after the third WAT (Left figure: GSAQ results before exercise; middle figure: GSAQ results immediately after the third WAT; right figure: GSAQ results 50 min after the third WAT).

The participants completed an adapted questionnaire to measure GI side effects before the test, immediately, and 50 min after the third WAT. The specific relevant GSAQ results are shown in Figure 6. The occurrence of gastrointestinal symptoms after enteric-coated sodium bicarbonate supplementation was minimal and no difference compared to placebo in the current study. At 180 min after enteric-coated sodium bicarbonate ingestion, the lowest GI symptoms were recorded with Protocol SES, with only 1 incidence where the participant reported mild side effects on the 10-point scale. The serial enteric-coated sodium bicarbonate supplementation regimen was well-tolerated. However, the greatest incidence of GI side effects was recorded with Protocol AES at 180 min after enteric-coated sodium bicarbonate ingestion. There were four reports of mild gastrointestinal symptoms after the enteric-coated sodium bicarbonate ingestion. Throughout the study protocol, none of the participants reported any meaningful gastrointestinal side effects (score >5 on a numeric rating scale).

**Figure 7:**
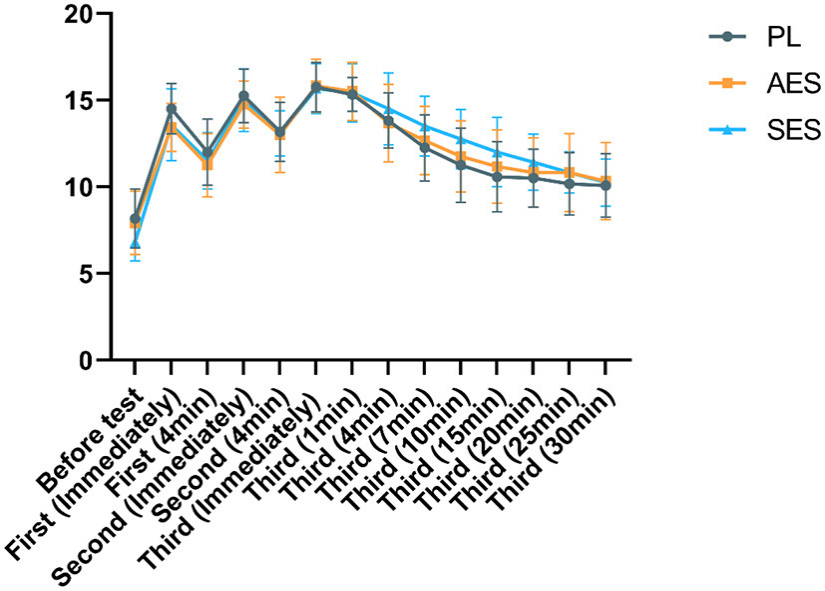
Ratings of perceived exertion (RPE) in three groups at each moment. Values are means ± SD. First (N min): N minute following the first WAT; Second (N min): N minute following the second WAT; Third (N min): N minute following the third WAT.

No significant differences were found for RPE between conditions [*F*_(2, 33)_ = 0.142, *P* = 0.868, η^2^ = 0.009]. SES had no strong effect on RPE (Figure 7). Furthermore, at almost all time points, the RPE for all ingestion protocols was likely to be substantially the same as those recorded in the PL trial.

### Relevant results of urine metabolomics

**Figure 8:**
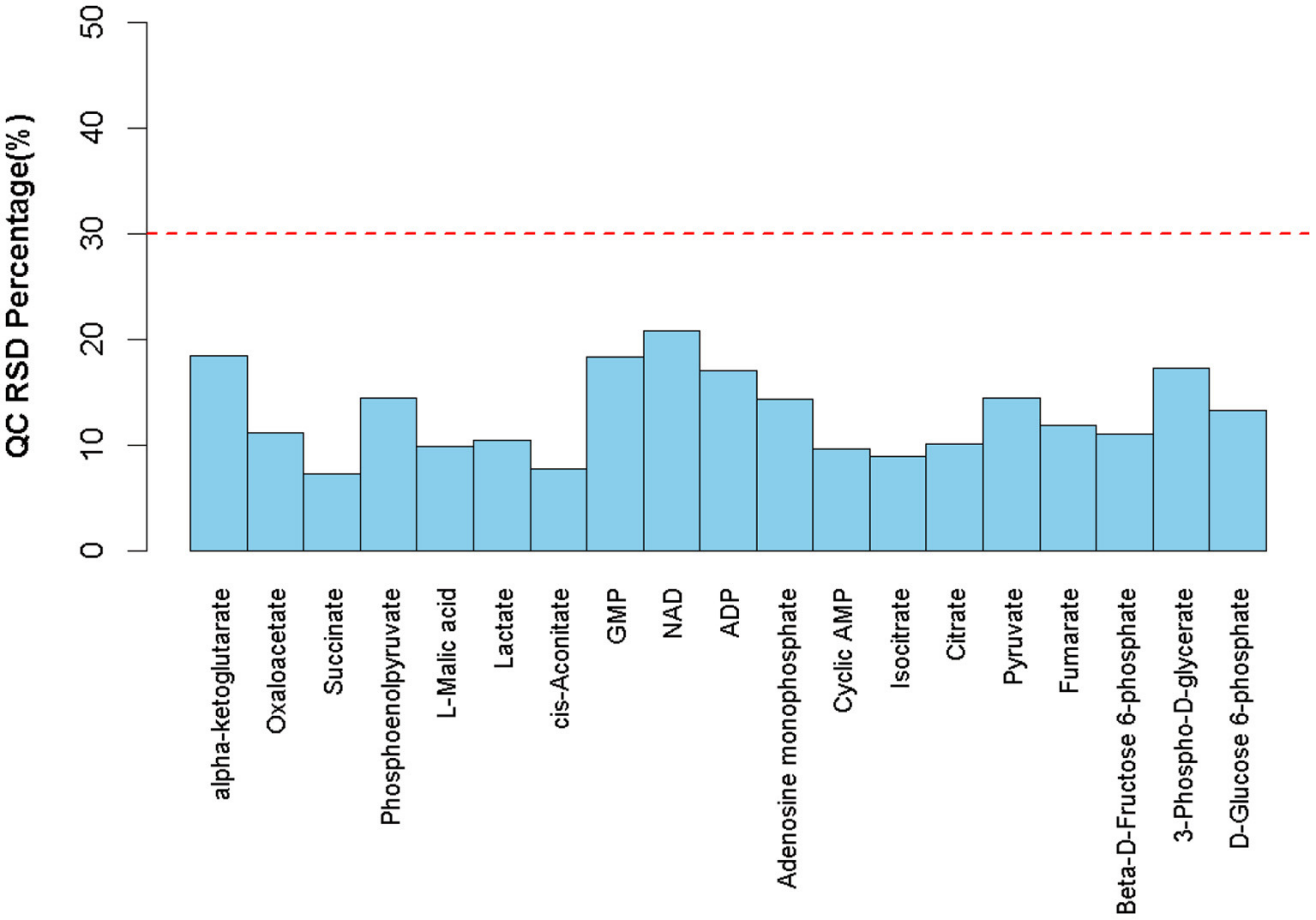
RSD distribution of QC samples.

All urine samples were equally mixed into QC samples, and the QC samples were used to evaluate the stability and reproducibility of the data. The RSD results of the analyte in QC samples are shown in Figure 8. Where RSD is <30% of energy metabolism, the data in the sample is stable and reliable.

**Figure 9:**
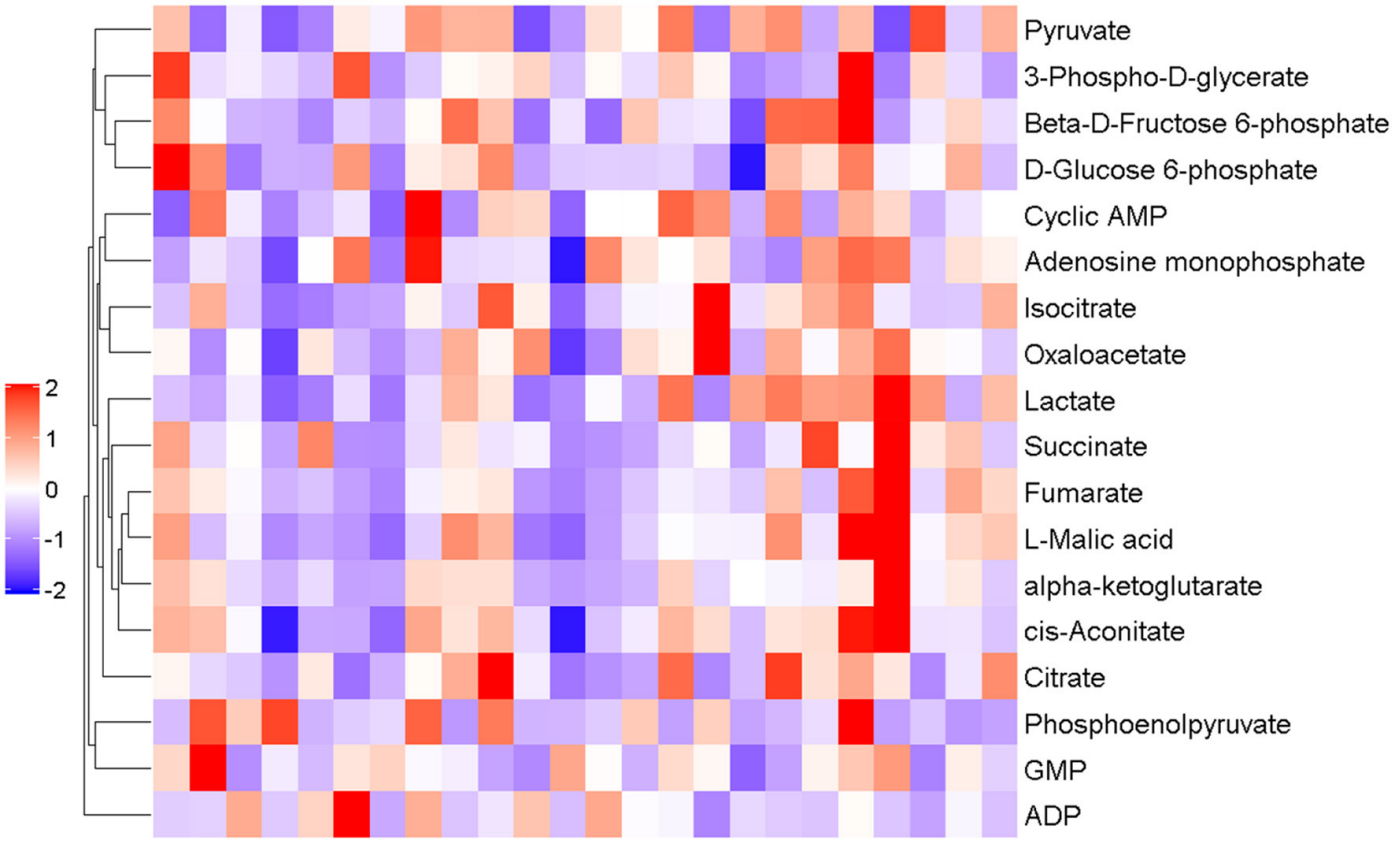
Results of hierarchical metabolite clustering at 50 min post-test. The horizontal coordinate indicates the sample and the vertical coordinate the intensity of metabolite expression; red indicates a positive correlation, purple indicates a negative correlation, and the darker color indicates higher metabolite intensity.

The results of hierarchical metabolite clustering are shown in Figure 9. Figure 9 visually depicts the trend of metabolic changes in human urine 50 min after the third WAT. When the selected candidate metabolites are reasonable and accurate, the same samples can be clustered into the same cluster. At the same time, the metabolites clustered in the same cluster have similar expression patterns and may be in a relatively immediate reaction step in the metabolic process. This indicates that different groups of interventions have significant effects on human urine.

**Figure 10:**
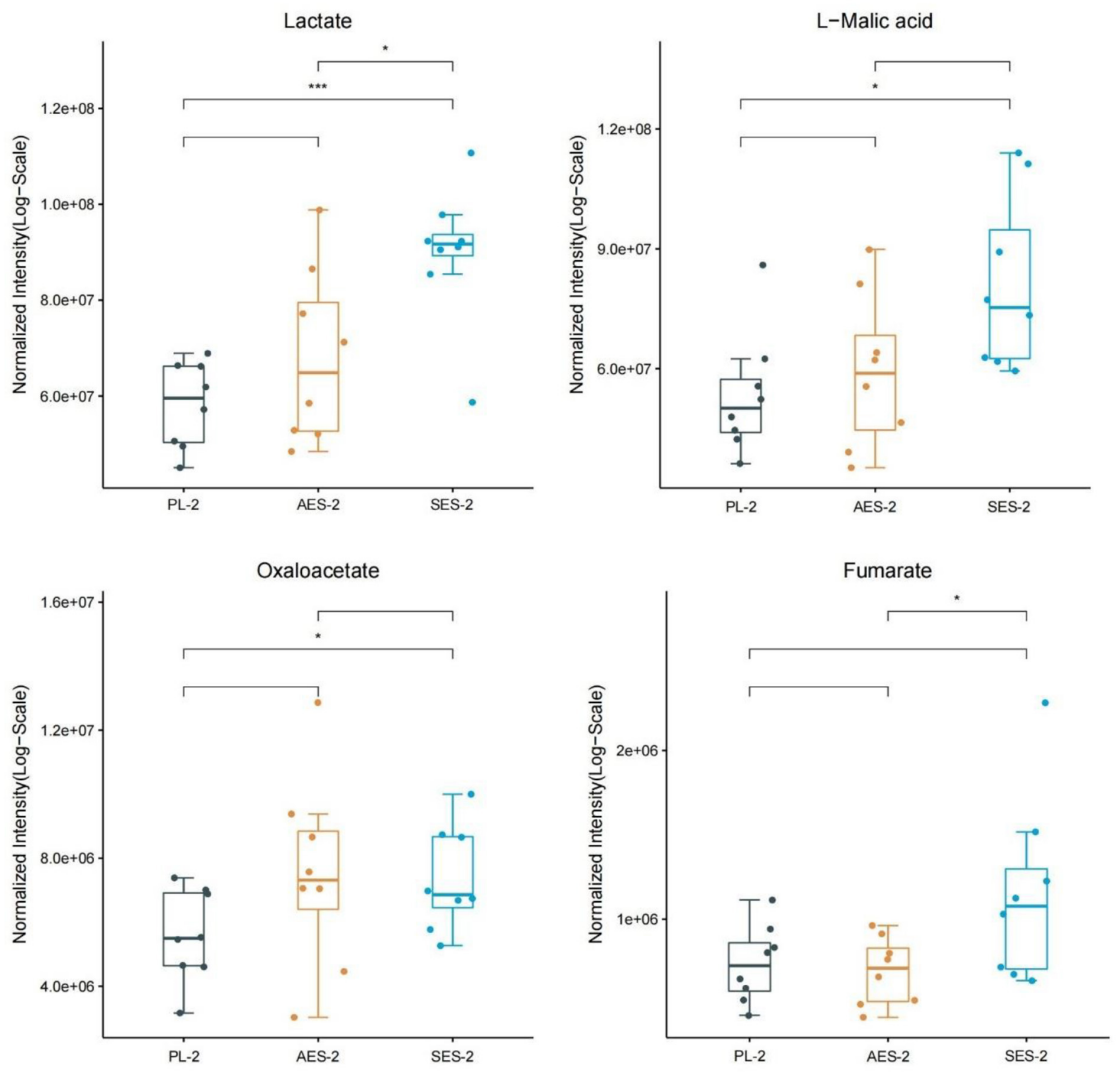
Metabolite expression trends between three groups. (PL, Placebo group; AES, acute enteric-coated sodium bicarbonate group; SES, serial enteric-coated sodium bicarbonate group; 2: 50 min after the third WAT). * Indicates *p* < 0.05; ** indicates *p* < 0.05; *** indicates *p* < 0.001.

Figure 10 shows the results and trends of metabolite screening. After screening and statistical analysis, 18 metabolites were screened in this study (Figure 9). A total of four differential metabolites were screened at 50 min post-WAT compared to the three sets of experiments, including lactate, L-Malic acid, oxaloacetate, and fumarate. In the 50 min after the third Wingate anaerobic test, the levels of lactate (*P* < 0.001), L-Malic acid (*P* < 0.05), and oxaloacetate (*P* < 0.05) were significantly higher in SES group than in the PL group. Compared with the AES group, the levels of lactate and fumarate in the SES group were significantly increased (*P* < 0.05).

## Discussion

The major finding of this study was that the relatively novel strategy of serial or acute enteric-coated sodium bicarbonate supplementation vs. placebo was implemented to investigate the effectiveness of the two different intervention strategies in improving anaerobic performance, physiological profile, and metabolomics.

### Anaerobic performance

According to the analyzed data, after three groups of intervention, MP (*p* < 0.05) and PP (*p* < 0.05) of SES all significantly improved compared with PL at the third WAT, implying that the effect of serial enteric-coated sodium bicarbonate supplementation significantly improves anaerobic capacity. On the contrary, acute enteric-coated sodium bicarbonate supplementation did not improve Wingate's performance. The serial supplementation of NaHCO_3_ is based on the assumption that chronic manipulation of pH-level may supply a protective effect on mitochondria, leading to improved mitochondrial function and consequently improved performance (20, 31). The effects of acute and chronic sodium bicarbonate supplementation on anaerobic capacity have been found in earlier studies (30, 32–35). In addition, there are many methods applied in studies of sodium bicarbonate, including the timing of supplementation, type of intake, and mode of exercise (22). The Wingate anaerobic test is commonly used to assess high-intensity performance and has been used in related studies to sodium bicarbonate. Some studies have explored the effect of sodium bicarbonate on Wingate's performance, but the results have been controversial (23, 33, 36). In a meta-analysis by Lopes-Silva et al. (37), nine studies (six acute and three chronic) examined the acute and chronic effects of sodium bicarbonate ingestion on Wingate test performance. The main findings of this meta-analysis were that, compared to placebo, acute ingestion of sodium bicarbonate did not improve either mean or peak power during the Wingate tests, regardless of the number of bouts performed. However, chronic ingestion of NaHCO_3_ improved peak and mean power in all bouts performed during the Wingate test relative to placebo (37). Overall, the meta-analysis of Lopes-Silva et al. was similar to our results. The values of MP and PP analyzed separately were not significantly different following acute enteric-coated sodium bicarbonate in our study but may have had some effect on anaerobic capacity. In SES protocol, the serial ingestion of enteric-coated sodium bicarbonate significantly increased only the MP and PP of the third WAT compared to placebo, with a significant effect on the third WAT. Although serial enteric-coated sodium bicarbonate supplementation did not significantly increase MP and PP for each WAT during Wingate testing compared to placebo (except for the third WAT), PP and MP values were higher in each WAT than in the other two groups. We speculate that this may be related to the intake of higher doses in other studies. As a result, it can be suggested that the ergogenic effects of enteric-coated sodium bicarbonate and training adaptations are more evident with serial rather than acute enteric-coated sodium bicarbonate supplementation.

### Blood lactate responses

Given that there was only a trend toward better lactate removal in the SES compared with the PL trial (not statistically significant). As shown in Figure 4, there was a significant decrease in BLA level only in SES over PL groups before the test (*p* < 0.05). SES seems to have shown a tendency to promote lactate clearance even before exercise. However, this finding requires further research. According to our results, no significant changes were found in the rise of BLA during the test compared to the PL group. Blood lactate concentrations were observed not to rise to their maximum in the first two Wingate tests. Presumably due to the duration of each Wingate test being 30 s and the need for longer exercise periods to facilitate the efflux of H^+^ and lactate from the active muscle. In addition, the duration of the test interval could also affect the rise in blood lactate. However, at the stage where lactate rises to its highest level, AES blood lactate values were higher than the other two groups, while SES had the lowest blood lactate values. The rationale for the ergogenic effects of bicarbonate is that the increase in extracellular pH and bicarbonate will enhance the efflux of lactate and H^+^ from the muscle cell (38). Based on previous studies in the literature, we observed similarly consistent results for blood lactate in the AES group but inconsistent results during serial ingestion. We speculate that this may be due to the high blood buffering capacity. SES has reached a relative equilibrium before testing and does not cause a particularly large blood reaction. In addition, we observed a faster decrease in BLA in both SES and AES groups compared to the PL group 15 min after the third WAT test. This is consistent with the results of other studies (36). This suggests that enteric-coated sodium bicarbonate may positively impact blood lactate clearance and be beneficial for anaerobic exercise performance.

While psychological HR indicators increased during exercise, no differences were reported between the three conditions (Figure 5). This is consistent with the fact that we did not find any reports indicating an improvement in heart rate recovery after exercise following sodium bicarbonate ingestion (36, 39).

### Perceptual responses

The results of GSAQ showed that gastrointestinal symptoms were mildly symptomatic in all three intervention groups (score <5). In particular, no gastrointestinal symptoms were observed before the test during SES supplementation. Although there were mild discomfort symptoms after the test, it is speculated that anaerobic exercise may be involved. Furthermore, we found that acute ingestion showed more gastrointestinal symptoms before exercise than serial ingestion. However, only mild gastrointestinal symptoms cannot be ruled out on the ability to exercise (40). The main limitation of sodium bicarbonate used for exercise is its gastrointestinal side effects, including nausea, diarrhea, and bloating (41). Saunders et al. (40) showed that athletes experiencing gastrointestinal distress are less likely to improve with sodium bicarbonate treatment. After acute sodium bicarbonate intake in a previous study, all participants reported gastrointestinal distress (41). Thus, it is necessary to identify protocols that alleviate the gastrointestinal symptoms of sodium bicarbonate. Enteric-coated sodium bicarbonate was supplemented in a multiday serial dose or acute dose regimen in our study. Thus, enteric-coated sodium bicarbonate supplementation may provide an alternative strategy to improve high-intensity exercise performance and alleviate GI symptoms associated with acute loading. This is consistent with previous studies (17). Given that enteric-coated sodium bicarbonate improves exercise performance among those with mild-to-moderate GI symptoms, the effects on exercise performance may be strengthened among those with more severe GI symptoms at the beginning of exercise (39).

We did not observe any differences in RPE between the three conditions. This is consistent with the results in other studies (34, 42). However, contrary to our results, some studies have shown that sodium bicarbonate supplementation during anaerobic exercise reduces RPE (43). We speculate that our findings may be since the anaerobic exercise was too intense and not subjectively felt by the participants to differentiate the RPE in a short time.

### Urine metabolomics

For human studies, urine can be collected in large quantities, non-invasively and continuously over a while, providing more complete information than blood. This assay of our study covers important metabolites in the TCA cycle, the glycolytic pathway, and oxidative phosphorylation. Based on this theory, targeted metabolomics was chosen for analysis and comparison in this study.

The lactate, L-Malic acid, and oxaloacetate levels of the SES group were significantly upregulated in the 50 min after the third Wingate anaerobic test compared to the PL group. Compared with the AES group, the levels of lactate and fumarate in the SES group were significantly increased. Our results indicated the presence of more lactate in urinary metabolites after serial supplementation with enteric-coated sodium bicarbonate, suggesting that serial enteric-coated sodium bicarbonate supplementation may be more beneficial for intramuscular lactate clearance. Glycolysis has long been known as a principal energy-generating pathway in tissues due to its high rates of ATP generation under anaerobic conditions (44). The metabolic pathway of glucose principally fuels lactate production through glycolysis and the pentose phosphate pathway. Studies have proved the effectiveness of urinary lactate as an indicator of anaerobic metabolism (45). *In vitro* studies have also suggested that increased extracellular ${\text{HCO}}_{3}^{-}$ concentration may contribute to lactate efflux from skeletal muscle (46). Given that lactate transport is stoichiometrically coupled with H^+^ transport (26), increased lactate efflux after sodium bicarbonate ingestion also indicates increased H^+^ efflux during exercise, reducing intramuscular H^+^ accumulation. Thus, we hypothesize that SES may enhance muscle buffering capacity and proton scavenging capacity more than AES and PL, induces glycolytic metabolism, and increases glycolytic ATP production.

L-Malic acid, oxaloacetate, and fumarate are intermediates in the TCA cycle. The TCA cycle is a common metabolic pathway in aerobic organisms, distributed in mitochondria, and is the metabolic link and final metabolic pathway for the three major nutrients (sugars, lipids, and amino acids). Generally, energy expenditure will be elevated with increasing exercise volume and intensity. The TCA cycle will be activated accordingly to oxidize and produce more ATP for the body (47), with a concomitant, marked increase in plasma TCA cycle intermediates (48). The SES group significantly increased the TCA cycle intermediate levels, including L-Malic acid, oxaloacetate, and fumarate. However, no changes in TCA cycle intermediate levels were found in AES. The magnitude of increased TCA cycle intermediates was larger in the SES group, presumably that SES better promoted a greater mitochondrial capacity for participants to generate energy during exertion (20, 30, 49).

In summary, after serial enteric-coated sodium bicarbonate supplementation, changes occurred in metabolism that involved the metabolism of lactate, L-Malic acid, oxaloacetate, and fumarate 50 min after exercise. This study suggests that serial enteric-coated sodium bicarbonate intake may accelerate lactate removal and induce the tricarboxylic acid cycle and glycolytic metabolism.

### Study limitations

As only blood lactate and post-exercise urine metabolomics were measured in this study, one can only speculate on the mechanisms that might be influenced by the serial and acute ingestion of enteric-coated sodium bicarbonate. However, determining the specific mechanism of effect is difficult, as it is unclear whether or to what extent enteric-coated sodium bicarbonate affects pH, ${\text{HCO}}_{3}^{-}$, or base excess concentrations. Therefore, further studies on the effects of serial and acute enteric-coated sodium bicarbonate ingestion are necessary to fully understand the underlying mechanisms contributing to exercise performance. In addition, our study used a relatively small sample of only healthy young men, which limits the generalizability to women and other populations. Finally, more research into serial vs. acute supplementation should be conducted to find out the correct supplementation protocol.

## Conclusion

Our results show that serial ingestion of enteric-coated sodium bicarbonate increases the content of MP and PP in the third Wingate anaerobic test, which may improve anaerobic exercise performance in healthy young men. Still, acute ingestion of enteric-coated sodium bicarbonate did not improve anaerobic exercise performance. Either with serial or acute supplementation doses, enteric-coated sodium bicarbonate produced fewer gastrointestinal symptoms and no difference compared to placebo, especially with no gastrointestinal side effects after serial supplementation. Moreover, acute supplementation and serial supplementation with enteric-coated sodium bicarbonate did not appear to induce different lactate reactions in the blood as previously reported. Still, it is possible that similar manifestations were observed to promote lactate clearance. Furthermore, serial enteric-coated sodium bicarbonate ingestion may cause changes in the metabolism of lactate, L-Malic acid, oxaloacetate, and fumarate 50 min after exercise, which presumably may promote the tricarboxylic acid cycle and lactate clearance.

## Data availability statement

The raw data supporting the conclusions of this article will be made available by the authors, without undue reservation.

## Ethics statement

The studies involving human participants were reviewed and approved by the Ethics Committee of Capital University of Physical Education and Sports approved the study (2021A42). The patients/participants provided their written informed consent to participate in this study.

## Author contributions

Conceptualization, investigation, writing—review and editing, supervision, project administration, and funding acquisition: HW. Methodology: NZ and XW. Software, formal analysis, data curation, and visualization: XK. Validation: YF. Resources: JW. Writing—original draft preparation: NZ and YF. All authors have read and agreed to the published version of the manuscript.

## Funding

The authors disclosed receipt of the following financial support for the research, authorship, and/or publication of this article: This work was supported by National Key Research and Development of China (No. 2018YFF0300603).

## Conflict of interest

Author JW was employed by Qingdao Shengbang Health Food Co. The remaining authors declare that the research was conducted in the absence of any commercial or financial relationships that could be construed as a potential conflict of interest.

## Publisher's note

All claims expressed in this article are solely those of the authors and do not necessarily represent those of their affiliated organizations, or those of the publisher, the editors and the reviewers. Any product that may be evaluated in this article, or claim that may be made by its manufacturer, is not guaranteed or endorsed by the publisher.
